# Management of inpatient chimeric antigen receptor T-cell therapy for relapsed/refractory B-cell malignancies: an analysis using the Japanese Diagnosis Procedure Combination database

**DOI:** 10.1007/s12185-025-04043-8

**Published:** 2025-08-06

**Authors:** Keisuke Tanaka, Hiroaki Kikuchi, Yoshihiro Umezawa, Takehiko Mori, Kiyohide Fushimi, Masahide Yamamoto

**Affiliations:** 1 Department of Hematology, Institute of Science Tokyo Hospital, 1-5-45 Yushima, Bunkyo-ku, Tokyo, 113-8510 Japan; 2 Department of Nephrology, Graduate School of Medical and Dental Sciences, Institute of Science Tokyo, Tokyo, Japan; 3 Department of Health Policy and Informatics, Graduate School of Medical and Dental Sciences, Institute of Science Tokyo, Tokyo, Japan

**Keywords:** Chimeric antigen receptor T-cell (CAR-T) therapy, Large B-cell lymphoma, B-Cell acute lymphoblastic leukemia, Cytokine release syndrome, Antifungal agents

## Abstract

Chimeric antigen receptor T-cell (CAR-T) therapy has shown remarkable efficacy in treating relapsed/refractory B-cell malignancies, as supported by real-world evidence (RWE). However, limited RWE exists on the management of adverse events during the perioperative period following CAR-T infusion. This study was conducted to obtain RWE on perioperative management using the Japanese Diagnosis Procedure Combination database, a comprehensive repository of Japanese health and medical service data. Between November 2019 and March 2022, 388 patients received CAR-T therapy. Of these, 312 had large B-cell lymphoma (LBCL) and 76 had B-cell acute lymphoblastic leukemia (B-ALL). The number of CAR-T infusions increased every 6-month interval, correlating with the rise in LBCL cases. Tocilizumab was administered for cytokine release syndrome in 56.1% of LBCL and 42.1% of B-ALL patients. Steroids were used for 22.9% and 81.3%, respectively. Prophylaxis for fungal infections was administered during CAR-T infusion in most LBCL and B-ALL patients. Treatment intensity was escalated in 2.8% of LBCL and 7.0% of B-ALL patients, and treatment for cytomegalovirus infection was initiated in approximately 7% of patients. This analysis elucidated perioperative management strategies based on patients’ medication histories.

## Introduction

CD19-directed chimeric antigen receptor T-cell (CAR-T) therapy has demonstrated significant therapeutic efficacy in treating relapsed or refractory (R/R) large B-cell lymphoma (LBCL) and B-cell acute lymphoblastic leukemia (B-ALL) [1–4]. Tisagenlecleucel (tisa-cel) became the first CAR-T therapy approved in Japan by the Pharmaceuticals and Medical Devices Agency in March 2019. Subsequently, axicabtagene ciloleucel (axi-cel) and lisocabtagene maraleucel (liso-cel) received approval in January 2021 and March 2021, respectively. The pivotal trials of CAR-T therapies demonstrated promising efficacy while also revealing therapy-specific adverse events, notably cytokine release syndrome (CRS) [1–4]. Subsequent retrospective analyses have further confirmed both efficacy and safety profiles [5–8]. However, these studies, predominantly from single or limited centers, may not fully represent the realities of routine clinical practice. Recent real-world evidence (RWE) from registry and healthcare insurance data [9–12] has shed light on the status of CAR-T therapy in clinical practice. Despite extensive RWE on the long-term efficacy, safety, and cost of CAR-T therapy, there is a notable lack of RWE regarding patient management during the perioperative period of CAR-T infusion. Therefore, this study aimed to elucidate the current status of perioperative management for CAR-T therapy using the Diagnosis Procedure Combination (DPC) database.

## Materials and methods

### Study design

We conducted a nationwide retrospective observational study in Japan to examine the epidemiology of patients with B-cell malignancies hospitalized for CAR-T therapy and to identify clinical management patterns. The Institutional Review Board of Institute of Science Tokyo University (M2000-788) approved our study, which was conducted in accordance with the principles of the Declaration of Helsinki.

This study used the DPC inpatient database, previously described in detail [13]. The DPC database comprehensively collects data from nearly all acute-care hospital inpatient admissions in Japan, including all CAR-T centers. The DPC database encompasses comprehensive patient information, including diagnoses, outcomes, medications administered, procedures conducted, and various disease-specific data points.

### Study subjects and data collection

We collected data on all inpatients who received CAR-T cell therapies (tisa-cel, axi-cel, and liso-cel) during the fiscal years 2019 to 2021. The fiscal year in Japan is from April of a year to March of the following year. Primary diagnoses were defined using International Classification of Diseases 10th revision (ICD-10) codes. ICD-10 codes C833 and C851 were classified as LBCL, while codes C910 and C913 were classified as B-ALL.

The following patient information was extracted from the DPC database: age, gender, admission date, discharge date, CAR-T cell infusion date, type of CAR-T cell products, and details of each drug used (name and administration date). Laboratory findings, imaging results, disease status, and adverse event severity data were unavailable in the DPC database.

### Lymphodepletion therapy

Data on the use of fludarabine (FLU), cyclophosphamide (CY), and bendamustine (BEN) were collected from day − 7 to day − 1, leading up to the day of CAR-T infusion (day 0). Drugs administered during this period were categorized as lymphodepletion (LD) therapy, while other drugs or no drugs were classified as "other."

### CAR-T therapy specific adverse events

CAR-T therapy can lead to specific adverse events, notably CRS and immune effector cell-associated neurotoxicity syndrome (ICANS). CRS typically manifests 2–3 days post-CAR-T infusion. Tocilizumab (TCZ) is the first-line treatment as per established therapeutic protocols [14, 15]. TCZ’s exclusive use for CRS means its administration signifies the onset of clinically significant CRS requiring intervention. If CRS symptoms persist despite TCZ, clinicians may administer steroids such as dexamethasone or methylprednisolone. ICANS frequently occurs after CRS, and steroids are typically the initial treatment for both ICANS and TCZ-resistant CRS. Given their early onset following CAR-T therapy, steroid use within 14 days of CAR-T administration is considered treatment for CRS/ICANS.

### Antifungal agents

In this analysis, we collected data on the use of antifungal agents (AFAs), including fluconazole (FLCZ), voriconazole (VRCZ), itraconazole (ITCZ), posaconazole (PSCZ), micafungin (MCFG), caspofungin (CPFG), and liposomal amphotericin-B (L-AMB). FLCZ was classified separately from other azole antifungal agents due to its widespread use as a universal prophylactic. Candin AFAs, including MCFG and CPFG, are also used for prophylaxis. Here, we grouped them together as candins. Patients using L-AMB or those treated with more than two drugs were classified under others.

Patients who initiated antifungal therapy concurrently with CAR-T treatment and later transitioned to azoles or incorporated additional agents post-CAR-T therapy were classified as having undergone treatment escalation. Changes between FLCZ and candins, or vice versa, were classified as a class switch. No escalation was defined as maintaining the current therapy, discontinuing treatment, or switching to FLCZ.

### Anti-cytomegalovirus agents

Data were collected on the use of ganciclovir (GCV), valganciclovir (VGCV), and foscarnet (FCN) as prophylactic and therapeutic agents for cytomegalovirus (CMV).

## Results

### Patient characteristics

In total, 388 patients received CAR-T therapy during the study period. Table 1 shows that the study included 312 patients with LBCL and 76 with B-ALL, with a slight male predominance observed in both cohorts. Liso-cel was administered in a minority of LBCL cases, while tisa-cel was used in the majority. FLU/CY served as the primary LD therapy, with BEN used in a small percentage (5.1%) of LBCL cases. Clinical trials of B-ALL did not include cases under 2 years of age [2]. However, the current study included five such cases. In contrast, LBCL clinical trials included patients over 80 years of age [1], while none of the patients in the current study exceeded 80 years old.

**Table 1:** Patients’ characteristics

	LBCL	B-ALL
	(*N* = 312)	(*N* = 76)
Age, median (range)	60 (19–78)	11 (1–25)
Male sex, *n* (%)	180 (57.7%)	41 (53.9%)
Lymphodepleting regimen, *n* (%)		
Cyclophosphamide + fludarabine	289 (92.6%)	70 (92.1%)
Bendamustine	16 (5.1%)	–
Other	7 (2.2%)	6 (7.9%)
CAR-T product, *n* (%)		
Tisa-cel	300 (96.2%)	76 (100%)
Liso-cel	12 (3.8%)	–

CAR-T, Chimeric antigen receptor T-cell; Tisa-cel, tisagenlecleucel; Liso-cel, lisocabtagene maraleucel; LBCL, large B-cell lymphoma; B-ALL, B-cell acute lymphoblastic leukemia

### Trends in CAR-T therapy cases

**Fig. 1:**
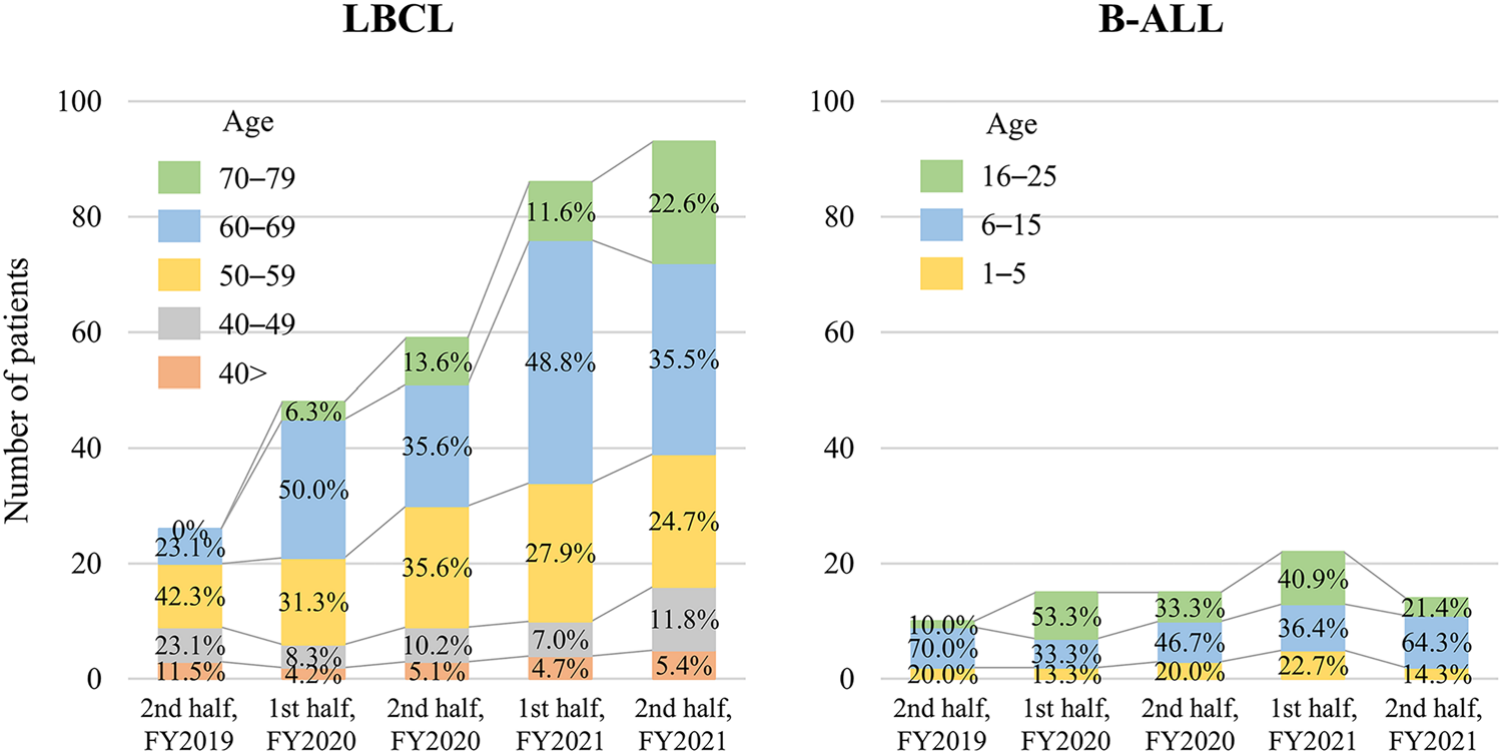
Changes in the number of CAR-T therapies by half-year. **A** LBCL, **B** B-ALL

Figure 1 shows the number of patients treated with CAR-T therapy every half-year period. The number of LBCL patients continued to rise, whereas the number of B-ALL patients remained stable. Initially, no patients aged 70 or older were in the LBCL group. However, by late FY2021, this age group comprised over 20% of total cases.

### CRS and ICANS

**Fig. 2:**
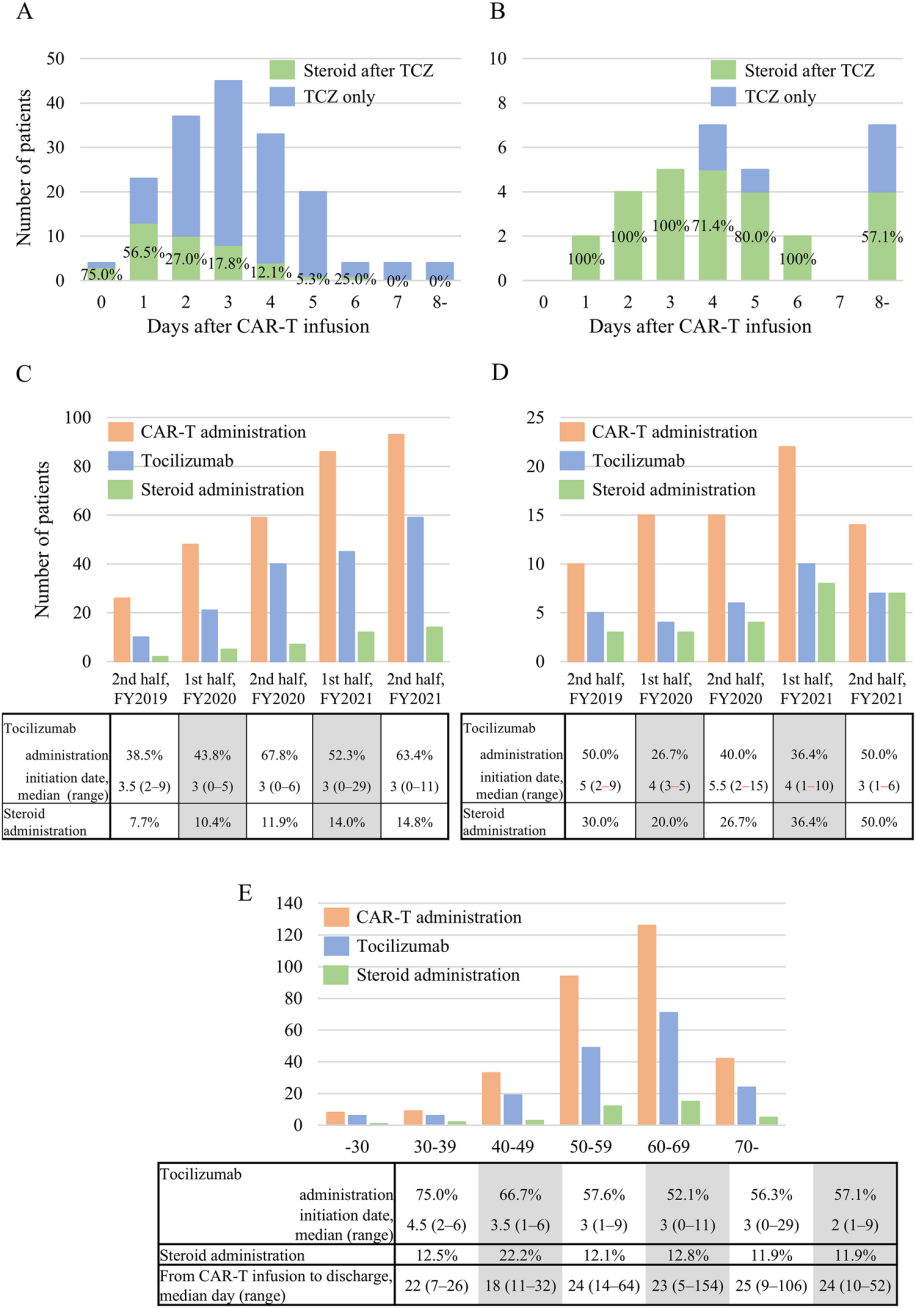
The number of cases using TCZ and steroids after CAR-T therapy. Number of cases using TCZ and steroids after CAR-T therapy in **A** LBCL and **B** B-ALL. Green bars indicate cases using both TCZ and steroids; blue bars indicate cases using only TCZ. Changes in the number of CAR-T therapies (orange bars), TCZ-used cases (green bars), and steroid-used cases (blue bars) by half-year. **C** LBCL, **D** B-ALL. **E** Number of CAR-T therapies (orange bars), TCZ-used cases (green bars), and steroid-used cases (blue bars) by age in LBCL patients

TCZ was administered to 175 patients (56.1%) with LBCL. The median time from CAR-T infusion to TCZ initiation was 3 days (range 0–29), with the vast majority (92.5%) commencing within 5 days post-infusion (Fig. 2A). Among patients who received TCZ, 40 subsequently required steroid treatment. The median time from TCZ initiation to steroid initiation was 2 days (range 0–6). A shorter interval between CAR-T infusion and TCZ initiation was associated with a higher likelihood of subsequent steroid treatment (Fig. 2A). Throughout the semi-annual periods of CAR-T therapy, the use of TCZ and steroids increased, while the timing of TCZ initiation remained consistent (Fig. 2C). No age-related differences were observed; however, patients in their 70 s had a shorter median time from CAR-T infusion to TCZ initiation (Fig. 2E).

In B-ALL, 32 (42.1%) of patients received TCZ, which was most frequently initiated on day 4 after CAR-T infusion. Most patients receiving TCZ also received steroids (Fig. 2B). Furthermore, the initiation of TCZ occurred earlier in the latter period, with steroids administered in approximately half of the cases (Fig. 2D).

Levetiracetam was administered prior to CAR-T cell infusion in 21.2% of patients with LBCL and 15.8% of those with B-ALL.

### Prevention and treatment of fungal infections

**Fig. 3:**
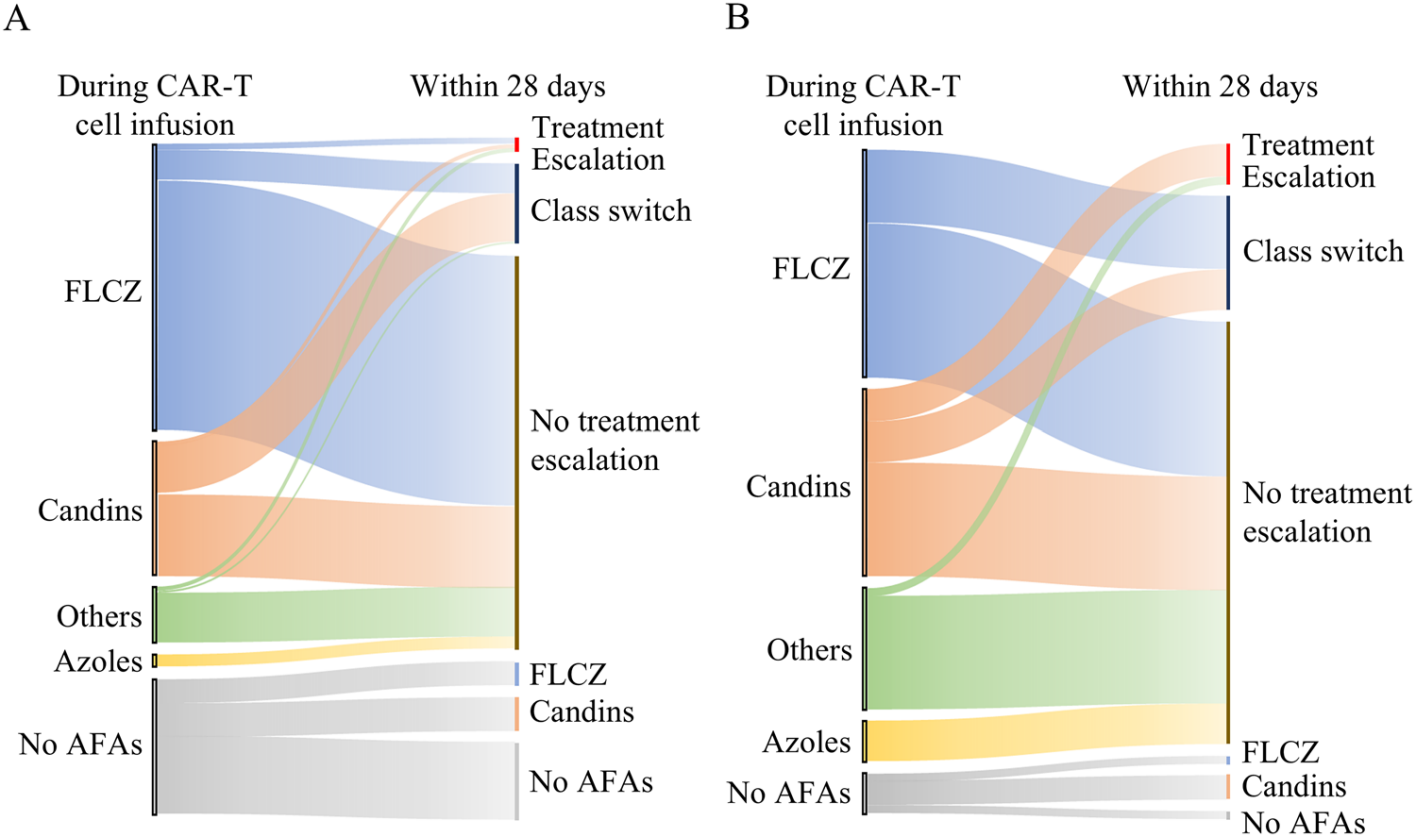
Changes in antifungal agents during the 28 days after CAR-T therapy. **A** LBCL, **B** B-ALL

Figure 3A, B shows changes in antifungal therapy within 28 days of CAR-T infusion. At infusion, 78.2% of LBCL patients and 93.4% of B-ALL patients received antifungal agents (AFAs), with fluconazole (FLCZ) being the most commonly administered in both groups. Among LBCL patients (Fig. 3A), of the 244 receiving AFAs at the start of CAR-T infusion, 197 were classified as no escalation, 40 as class switch, and only 7 (2.8%) experienced treatment escalation. Similarly, in B-ALL (Fig. 3B), 52 of 71 patients (73.2%) maintained their initial treatment regimen, while only 5 cases showed clear treatment escalation. No patients with LBCL or B-ALL who did not receive AFAs at the time of CAR-T infusion were treated with azoles or other antifungal agents.

### Prevention and treatment of cytomegalovirus

Most patients were not receiving anti-CMV medications when CAR-T cells were infused (Table 2). Among these patients, only 21 LBCL (7.0%) and 5 B-ALL (6.7%) initiated anti-CMV treatment within 28 days of receiving CAR-T therapy. In most of them, anti-CMV agents were started after day 14. Conversely, most patients who received anti-CMV agents at CAR-T cell infusion discontinued them within 28 days. In both LBCL and B-ALL, patients who received corticosteroids within two weeks after CAR-T cell infusion were more likely to undergo anti-CMV treatment compared to those who did not. However, the differences were not statistically significant (LBCL: 15.8% vs. 6.7%, *p* = 0.096; B-ALL: 15.4% vs. 4.1%, *p* = 0.174).

**Table 2:** Use of anti-CMV medication after CAR-T infusion

During CAR-T infusion	Within 28 days after CAR-T infusion
LBCL	
No therapy (*n* = 300)	
	No therapy (*n* = 279, 93.0%)
	Initiate anti-CMV agents (*n* = 21, 7.0%)
Anti-CMV agents (*n* = 12)	
	Stop anti-CMV agents (*n* = 9, 75%)
	Continue same anti-CMV agents (*n* = 2, 16.7%)
	Change to other anti-CMV agent (*n* = 1, 8.3%)
B-ALL	
No therapy (*n* = 75)	
	No therapy (*n* = 70, 93.3%)
	Initiate anti-CMV agents (*n* = 5, 6.7%)
Anti-CMV agents (*n* = 1)	
	Stop anti-CMV agents (*n* = 1, 100%)

CAR-T, Chimeric antigen receptor T-cell; LBCL, large B-cell lymphoma; B-ALL, B-cell acute lymphoblastic leukemia; CMV, cytomegalovirus

### Length of hospital stay and death during hospitalization

The median hospital stay after CAR-T infusion was 25 days (range 6–155) for LBCL patients and 32 days (range 1–180) for B-ALL patients. Hospital stays in LBCL decreased with more recent treatment dates, while B-ALL showed no such trend (data not shown). Hospital stay duration showed no significant variations related to patient age or CRS onset timing (data not shown).

All deaths during hospitalization occurred in cases of LBCL, with three occurring very early within 10 days of treatment, and the remaining six occurring 40 days or more after treatment.

## Discussion

Immunotherapy has revolutionized cancer treatment, with CAR-T cell therapy emerging as a cornerstone in managing B-cell malignancies. The efficacy of CAR-T therapy has been confirmed not only in pivotal studies but also in RWE [1–10]. While CAR-T therapy is now an established treatment modality in daily practice, RWE for perioperative management remains limited to date. Our analysis clarifies this aspect of RWE using Japanese DPC data.

Approximately 400 patients received CAR-T therapy in this analysis, with the majority treated using tisa-cel. This represents not only the first RWE from Japan but also one of the largest datasets on tisa-cel use globally. As previously reported [16, 17], CAR-T therapy has been steadily increasing year after year, while the number of B-ALL cases has remained relatively stable. In this study, no deaths occurred and few serious fungal infections were observed among B-ALL patients. Additionally, the study included patients younger than 2 years of age, who were not included in the clinical trial. This suggests that adverse events are not the reason for the lack of increase in case numbers, and that the upper age limit of 25 years is the responsible factor. The rising incidence of LBCL cases, especially among individuals over 70, has primarily driven the annual increase in CAR-T therapy cases. Recently, cases involving individuals over 70 years old have exceeded 20% of all reported cases (Fig. 1A). Therefore, CAR-T therapy is considered suitable for both younger and older patients, and its use is expected to continue increasing.

Regarding CRS, we identified RWE based on the history of TCZ use. While previous RWE studies have described the frequency of these events (both all grades and grade 3 or higher), the details of therapeutic interventions have not been well documented [9–11]. In this study, 56.1% of patients with LBCL and 42.1% of those with B-ALL developed cytokine release syndrome requiring therapeutic intervention. Previous reports on tisa-cel indicated that the frequency of TCZ use ranged from 13 to 58% in LBCL [6, 7, 11, 18–21] and 25 to 45% in B-ALL [22–24]. Compared with the Japanese data, the rate of TCZ use was comparable in both LBCL and B-ALL, supporting the reliability of the DPC data [21, 24]. In contrast, there was a discrepancy in steroid use compared to previous reports. In LBCL cases, steroids were administered in the early phase following TCZ administration, suggesting their use was targeted at managing CRS or ICANS. The previously reported data were derived from only five institutions, which may have contributed to the observed difference [21]. Therefore, the present study are considered to more closely reflect real-world data. The rate of steroid use in patients with B-ALL was higher in our study compared to real-world data from Japan [24]. Our study included cases treated in a more recent period than those in previous reports, and a change in the approach to steroid administration over time may be one of the factors contributing to this difference. These results also suggest that, compared to LBCL, CRS is more challenging to control using TCZ as a single agent.

Comprehensive RWE on managing infectious complications in CAR-T therapy is limited, particularly regarding fungal prophylaxis, where comprehensive reports are lacking. Approximately 15% of patients undergoing CAR-T therapy develop infections within 28 days, with bacterial infections being most prevalent, although fungal and viral infections have also been reported [7, 25]. Nevertheless, specific preventive and therapeutic measures for fungal and viral infections have remained elusive until recently and we primarily focused on the analysis of fungal and viral infections. In our analysis, most patients received AFAs, primarily FLCZ or candins, at the initiation of CAR-T therapy. This practice aligns with the American Society for Transplantation and Cellular Therapy (ASTCT) guidelines, which recommend prophylaxis with FLCZ or an echinocandin in routine clinical practice [26]. Notably, our analysis revealed that, regardless of prophylaxis, few cases required escalation of treatment intensity, and the incidence or exacerbation of fungal infections shortly after CAR-T infusion was minimal. For viral infections, we focused on CMV prophylaxis and treatment. Few patients with LBCL or B-ALL received medication prior to CAR-T therapy. Approximately 7% of cases initiated anti-CMV treatment during hospitalization (Table S1). Given that CMV reactivation after CAR-T therapy has been observed in approximately 20% of cases [25, 27], the 7% incidence of cases requiring therapeutic intervention in this study reflects actual clinical practice and should not be considered a rare adverse event.

The duration of hospitalization was also analyzed. B-ALL patients experienced significantly longer hospital stays compared to those with LBCL. For B-ALL, hospitalization duration remained consistent regardless of treatment timing. However, LBCL patients experienced shorter hospital stays in more recent treatment periods, with the median length decreasing to 22 days in the most recent timeframe. Previous reports indicated a median hospitalization length of 14–21 days [5, 10, 11], potentially due to differences in healthcare insurance systems. We also examined differences in hospitalization duration for LBCL based on age, presence or absence of CRS, and disease onset timing, but found no significant variations.

A limitation of this study is the lack of comprehensive clinical data beyond survival outcomes, restricting the analysis to information derived solely from medication usage. However, we successfully clarified the frequency of CRS requiring therapeutic intervention and the current status of prevention and treatment for fungal and CMV infections in Japanese clinical practice. In particular, there have been no comprehensive reports on fungal prophylaxis. The study demonstrated that fungal prophylaxis was commonly administered in routine practice, resulting in fewer complications from severe fungal infections among recipients. Given no serious fungal complications were observed in LBCL patients without prophylaxis, fungal prophylaxis may not be necessary for all patients receiving CAR-T therapy. This analysis of drug usage in the DPC database provides valuable insights for routine clinical practice. Combining these findings with registry data in the future is expected to further enhance the quality of RWE.

## Data Availability

The data that support the findings of this study are available from the corresponding author upon reasonable request.
